# Hot Pack in Puerperal Eclampsia

**Published:** 1882-11

**Authors:** 


### HOT PACK IN PUERPERAL ECLAMPSIA.

For the cure of puerperal eclampsia either in the puerperium or the last months of pregnancy, active diaphoresis alone, induced by a hot bath, 40 to 45°C, followed by the pack, is all sufficient. The bath must not be prolonged over one-half hour, and two to three hours suffices for the envelopment in the pack. This method properly carried out, according to Brens, will also cause œdema and albuminuria to disappear without interruption of pregnancy.—Brens, *Arch. f. Gyn. xix, p.* 218.

